# Multicomponent Mechanical Characterization of Atherosclerotic Human Coronary Arteries: An Experimental and Computational Hybrid Approach

**DOI:** 10.3389/fphys.2021.733009

**Published:** 2021-09-07

**Authors:** Su Guvenir Torun, Hakki M. Torun, Hendrik H. G. Hansen, Giulia Gandini, Irene Berselli, Veronica Codazzi, Chris L. de Korte, Antonius F. W. van der Steen, Francesco Migliavacca, Claudio Chiastra, Ali C. Akyildiz

**Affiliations:** ^1^Department of Biomedical Engineering, Erasmus Medical Center, Rotterdam, Netherlands; ^2^School of Electrical and Computer Engineering, Georgia Institute of Technology, Atlanta, GA, United States; ^3^Department of Medical Imaging, Radboud University Medical Center, Nijmegen, Netherlands; ^4^Laboratory of Biological Structure Mechanics, Department of Chemistry, Materials and Chemical Engineering “Giulio Natta,” Politecnico di Milano, Milan, Italy; ^5^Physics of Fluids Group, TechMed Centre, University of Twente, Enschede, Netherlands; ^6^PolitoBIOMed Lab, Department of Mechanical and Aerospace Engineering, Politecnico di Torino, Turin, Italy; ^7^Department of Biomechanical Engineering, Delft University of Technology, Delft, Netherlands

**Keywords:** atherosclerosis, Bayesian optimization, coronary artery, finite element analysis, inflation test, material constant, ultrasound, Yeoh model

## Abstract

Atherosclerotic plaque rupture in coronary arteries, an important trigger of myocardial infarction, is shown to correlate with high levels of pressure-induced mechanical stresses in plaques. Finite element (FE) analyses are commonly used for plaque stress assessment. However, the required information of heterogenous material properties of atherosclerotic coronaries remains to be scarce. In this work, we characterized the component-wise mechanical properties of atherosclerotic human coronary arteries. To achieve this, we performed *ex vivo* inflation tests on post-mortem human coronary arteries and developed an inverse FE modeling (iFEM) pipeline, which combined high-frequency ultrasound deformation measurements, a high-field magnetic resonance-based artery composition characterization, and a machine learning-based Bayesian optimization (BO) with uniqueness assessment. By using the developed pipeline, 10 cross-sections from five atherosclerotic human coronary arteries were analyzed, and the Yeoh material model constants of the fibrous intima and arterial wall components were determined. This work outlines the developed pipeline and provides the knowledge of non-linear, multicomponent mechanical properties of atherosclerotic human coronary arteries.

## Introduction

Atherosclerosis is a systemic progressive disease of arteries, characterized mainly by arterial wall thickening due to plaque tissue formation (Lusis, 2000). Plaque rupture in atherosclerotic coronaries is highly critical as this triggers thrombosis, a main cause of acute myocardial infarction (Virmani et al., 2009). Accurate assessment of coronary plaque rupture risk is crucial to prevent fatal coronary events. However, the current assessment criteria are far from being optimal. Thus, there is a need for better risk assessment tools (Akyildiz et al., 2018).

Previously, high mechanical stresses induced by blood pressure were shown to associate with plaque rupture (Richardson, 1989; Cheng et al., 1993). Plaque stress analyses, usually performed with computational techniques such as finite element (FE) modeling, have the great potential to further optimize the plaque rupture risk assessment. In these analyses, the material properties of the individual structural components of atherosclerotic coronary arteries, especially of the fibrous intima, are critical as they were shown to greatly influence the stress results (Akyildiz et al., 2011). However, the material property information of atherosclerotic human coronaries is lacking greatly (Akyildiz et al., 2014) as collecting, preparing, and mechanical testing of atherosclerotic human coronaries are challenging (Jankowska et al., 2015).

A few studies investigated the material properties of atherosclerotic human coronaries (Kural et al., 2012; Karimi et al., 2013a,b, 2017a,b; Jankowska et al., 2015). These studies, where uniaxial or biaxial tensile tests were performed on intact artery strips (i.e., the individual layers/components not separated), provided aggregate tissue properties rather than the properties of the individual tissue components. Recently, our group (Akyildiz et al., 2016) developed a hybrid experimental and numerical framework, based on the inverse FE modeling (iFEM) technique, for the multicomponent material characterization of atherosclerotic arteries and tested its feasibility on atherosclerotic porcine iliac arteries. The framework comprised (i) inflation tests, which mimic the physiological multiaxial loading of the arteries more closely than commonly used tensile tests; (ii) high-resolution, ultrasound-based local tissue deformation measurements; (iii) histology-based FE modeling; and (iv) grid search optimization algorithm.

In the current study, we further advance our framework and use it to characterize the multicomponent material properties of atherosclerotic human coronary arteries. The advancements include (1) high-resolution magnetic resonance imaging (MRI) for detailed multicomponent geometrical information of arteries, (2) use of Yeoh material model to represent the non-linear tissue behavior, and (3) integration of a machine learning-based, cost-efficient Bayesian optimization (BO) technique. To the best of our knowledge, this work is the first to assess the multicomponent material properties of atherosclerotic human coronaries from physiological, intact artery testing.

## Materials and Methods

**FIGURE 1:**
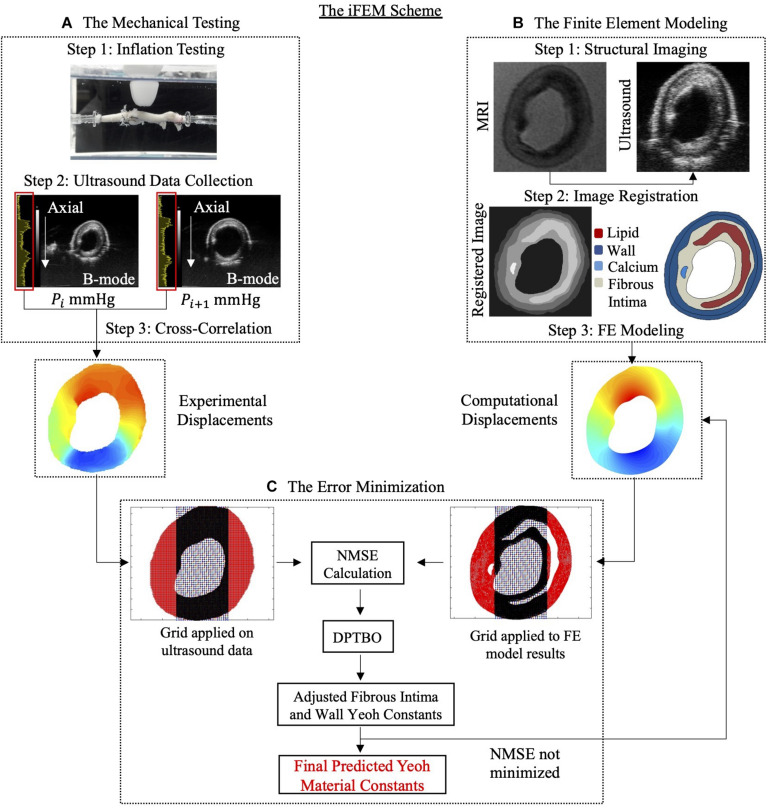
The inverse finite element modeling (iFEM) scheme composed of three main parts: mechanical testing **(A)**, finite element modeling **(B)**, and error minimization **(C)**. DPTBO, deep partitioning tree based Bayesian optimization; FE, finite element; MRI, magnetic resonance imaging; NMSE, normalized mean square error; RF, radiofrequency.

The iFEM approach was used to characterize the non-linear material behavior of the fibrous intima and the arterial wall components of atherosclerotic human coronaries. The main steps of the method were: (A) mechanical testing, (B) FE modeling, and (C) error minimization process (Figure 1).

### Mechanical Testing

In the first part of the iFEM scheme, the inflation testing was combined with a high-resolution preclinical ultrasound imaging system and cross-correlation technique to obtain experimental deformations of the atherosclerotic human coronary arteries.

#### Coronary Artery Collection and Preparation

Five atherosclerotic human coronary arteries were collected post-mortem, snap frozen, and stored at −80°C until the day of testing. Two of the arteries were right coronaries, whereas the others were left anterior descending coronary arteries. Prior to the inflation tests, the arteries were thawed at room temperature and the connective tissues outside the arteries were removed carefully. The side branches of the arteries were closed with surgical suture to prevent leakage during the tests. The collection and use of human coronaries for this study were approved by the Medical Ethics Committee of Erasmus Medical Center in Rotterdam.

#### Inflation Testing Setup and Testing Protocol

The inflation test setup consisted of a tissue bath, a heating unit, a syringe pump (Pump 11 Elite, Harvard Apparatus, Holliston, MA, United States), and a digital pressure manometer (GDH200, Greisinger Electronic, Regenstauf, Germany). During the inflation tests, the cannulated arteries were submersed in phosphate buffered saline (PBS) solution at 37°C in the tissue bath. While one end of the arteries was connected to the syringe pump, which injected PBS in to the arteries, the other end was connected to the pressure manometer. Prior to the experiments, the arteries were preconditioned by applying an intraluminal pressure of 80 mmHg and a longitudinal (in the direction along the long axis of the artery) stretch of 20% for 10 cycles. To prevent a possible buckling and to reach the approximately *in situ* length, the arteries were tested at 20% longitudinal pre-stretch.

During the inflation tests, quasi-static pressure steps of 10 mmHg were applied up to 120 mmHg. At each pressure step, the arteries were imaged with an ultrasound system (Vevo 2100, FUJIFILM, Visual Sonics Inc., Toronto, ON, Canada). The integrated motor of the system enabled acquiring 2D transversal scans of the arteries at multiple locations during inflation. A high-frequency probe (MS550S, fc = 40 MHz) was used for the ultrasound scans. This way, transversal, 2D B-mode images and radiofrequency (RF) data were collected at approximately every 0.137 mm along the longitudinal direction of the arteries. The collected RF data were subsequently used for the cross-correlation technique (Lopata et al., 2009; Akyildiz et al., 2016) to determine the local tissue deformation induced by the inflation.

#### Measurements of Local Tissue Deformation

Local tissue deformation (in terms of axial – along the ultrasound wave direction – and lateral – perpendicular to the ultrasound wave direction – displacements, referring to 2D cross-sectional images) were measured using an iterative coarse-to-fine block-matching algorithm (Lopata et al., 2009) from ultrasound RF data. In iteration #1, estimates of the displacements were obtained on a coarse grid by determining 2D normalized cross-correlation functions for templates of 2D demodulated RF data from one pressure step with large kernels of data from a subsequent pressure step. The difference in position between the cross-correlation functions’ peaks and the centers of the templates directly provided the displacement values in 2D. Then, the coarse displacements were filtered using a 2D median filter (size: 5×5). The filtered displacements served as initial guesses for displacement estimation on a finer scale in iteration #2. In this iteration, smaller templates and search kernels were used for more local displacement estimation. After iteration #2, displacements were again filtered with a 2D median filter (size: 41 × 41). In the final iteration, #3, the finest, most local, displacements were obtained. Again, the displacements estimated in the previous iteration were used as initial offset for the search kernels, and again template and kernel sizes were smaller. For highly accurate displacement estimation, the raw RF data instead of the demodulated RF data were used for the block-matching operation in this final iteration. Furthermore, the peak positions of the normalized cross-correlation functions were estimated on a subsample scale by 2D cubic interpolation of the cross-correlation peak (Saris et al., 2018). No filtering was performed after the last iteration. Displacements from the lowest-pressure step to the highest-pressure step were accumulated using bilinear interpolation. For more details on this accumulation step, see Hansen et al. (2014). The specific kernel, grid, and template sizes are given in Table 1. The algorithm provides axial and lateral displacements as an output. For the iFEM scheme, only the axial displacements were utilized due to relatively higher noise in lateral direction (Akyildiz et al., 2016).

**TABLE 1:** Coarse-to-fine displacement algorithm settings.

**Iteration**	**Demodulated RF**	**Template sizes (μm × μm)**	**Search kernel sizes (μm × μm)**	**Grid spacing (μm × μm)**
#1	Yes	481.3 × 412.5	1684.4 × 962.5	481.3 × 27.5
#2	Yes	240.6 × 247.5	360.9 × 522.5	27.1 × 27.5
#3	No	120.3 × 137.5	144.4 × 192.5	27.1 × 27.5

### Finite Element Modeling

In the second part of the iFEM scheme, the inflation testing of the arteries was simulated using FE models to obtain the local tissue deformation computationally. The geometrical information of the arteries was obtained by the ultrasound registered MRI images.

#### Plaque Morphology by Magnetic Resonance Imaging

**FIGURE 2:**
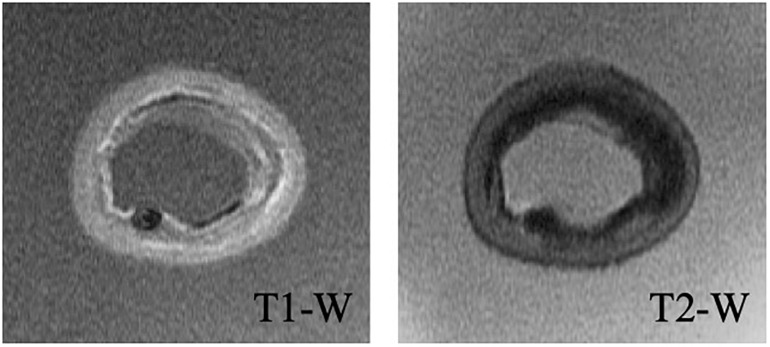
Representative T1- and T2-weighted MRI images of cross-section #9.

A high-field, 7-Tesla, pre-clinical MRI system (Discovery MR901, General Electrics, Fairfield, CT, United States) was used to obtain the detailed geometry of the lipid, calcium, fibrous intima, and arterial wall (consisting of the media and adventitia layers) components. T1- and T2-weighted sequences (Figure 2) were used to image the components, while the artery was submerged in PBS solution during the imaging. The repetition time and echo time were 17.48 and 3.35 ms for the T1-weighted, and 2500 and 66 ms for the T2-weighted sequences, respectively. The flip angle was 90° in both sequences. Both the slice thickness and interslice spacing were chosen as 0.5 mm. The obtained T1- and T2-weighted MR images had a spatial resolution of 49 μm × 49 μm.

Lipid and calcium components were identified as the hypointense (dark) regions in T2-weighted images (Shinnar et al., 1999; Serfaty et al., 2001). The T1-weighted images helped to differentiate calcium from the lipid component (Tang et al., 2009) as the calcium appeared hypointense in T1-weighted images (Shinnar et al., 1999). The fibrous intima appeared as hyperintense (bright) on T2-weighted images (Martin et al., 1995). T2-weighted images were also used to determine the arterial wall components, the adventitia and the media, which appeared as hypo and hyperintensity regions, respectively (Serfaty et al., 2001).

#### Cross-Section Selection

Multiple cross-sections from the five tested arteries were selected for further analyses. The selection was based on the location, plaque size, and the ultrasound and MRI image qualities of the cross-sections. The cross-sections close to a side branch or a cannula were not included. The selected cross-sections were required to have clear lumen and external elastic lamina border on MRI and ultrasound images. In addition, both images had to be free of any artifact due to air bubbles in PBS. Multiple cross-sections from the same artery were selected only if these cross-sections were at least 1.5 mm apart from each other. Application of these strict selection criteria resulted in a set of 10 cross-sections, where the number of cross-sections per each artery ranged from 1 to 4 (Table 2).

**TABLE 2:** iFEM predicted Yeoh constants for fibrous intima and wall, and their respective NMSE values.

		**Fibrous intima (kPa)**			**Wall (kPa)**			**NMSE (%)**
		**c_1_**	**c_2_**	**c_3_**	**c_1_**	**c_2_**	**c_3_**	
Artery 1	CS 1	0.10	0.04	0.15	6.17	0.04	298.93	1.61
	CS 2	0.11	0.29	0.47	15.04	4.39	237.01	2.15
Artery 2	CS 3	6.64	0.11	0.15	7.40	−5.60	187.62	6.62
Artery 3	CS 4	2.03	57.42	1.56	24.92	−141.80	467.32	9.56
Artery 4	CS 5	0.66	4.12	0.12	14.11	−1.63	38.39	23.01
	CS 6	3.93	−0.59	3.76	5.62	−0.59	103.36	6.85
	CS 7	0.33	1.10	0.19	0.10	112.57	193.34	17.74
	CS 8	13.12	−5.86	7.42	2.03	−3.52	7.42	8.73
Artery 5	CS 9	0.10	0.60	0.26	0.80	0.05	674.04	6.98
	CS10	18.99	−96.68	289.37	0.42	0.59	2.30	21.60

*iFEM, inverse finite element modeling; NMSE, normalized mean square error.*

Since MRI images were used for tissue composition and ultrasound data for the experimental tissue deformation measurements, the data sets from these imaging modalities had to be co-registered. The MRI and ultrasound images corresponding to a cross-section were identified by using the cannulas and side branches as landmarks, which were visible in both imaging modalities. Based on the distance of the cross-section from these landmarks, the correct image in both modalities was identified.

#### Image Segmentation, and MRI and Ultrasound Co-registration

**FIGURE 3:**
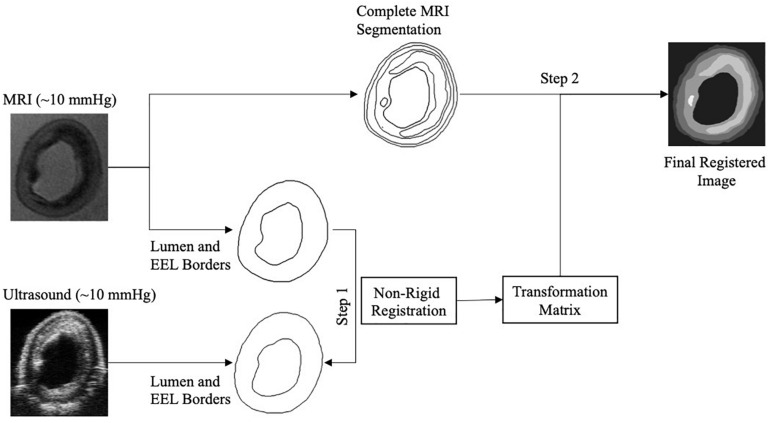
Image registration composed of the registration of lumen and external elastic lamina (EEL) borders of the magnetic resonance imaging (MRI) to the ultrasound to obtain the transformation matrix (Step 1), and the application of the transformation matrix to the complete segmented MRI image (Step 2).

Once the correct images of a cross-section from the ultrasound and MRI data sets were selected, these two images had to be co-registered. For the co-registration, approximately 10 mmHg pressurized reference ultrasound and MRI images were used. A custom-built script within the MevisLab Software (version 2.7, MeVis Medical Solutions, AG, Fraunhofer Institute for Digital Medicine MEVIS, Bremen, Germany), the open-source software ITK-Snap (Yushkevich et al., 2006), and *elastix* (Klein et al., 2010) were utilized. The developed MevisLab tool was used to adjust the spatial resolution of the MRI to match the one of the ultrasound data and to perform a rigid registration. Then, ITK-Snap was used to delineate the lumen and the external elastic lamina (the border between the media and the adventitia) on the MRI and the ultrasound images of the selected cross-sections. Subsequently, MRI segmentations were co-registered to ultrasound segmentations through non-rigid deformation using *elastix*. This provided a co-registration transformation matrix. Then, MRI images were further segmented for the lipid, calcium, fibrous intima, and arterial wall components by using ITK-Snap. The obtained transformation matrix was applied on the fully segmented MRI image to transfer the geometries of the components to the ultrasound images (Figure 3). This final registered image obtained with this protocol was used to create the multicomponent geometry of the selected cross-sections for the FE models.

#### Details of Finite Element Models

The 2D FE models were built in ABAQUS (version 2016, Dassault Systèmes, Vélizy-Villacoublay, France). The atherosclerotic coronary artery geometries were discretized with quadrilateral (CPE4H) and triangular (CPE3H) hybrid, plane strain elements. Following the mesh refinement study, the minimum global element size was defined as 0.035 mm, and the average number of nodes was approximately 19k ± 5k. The models were used to simulate the intraluminal pressurization during the inflation tests by applying pressures of 80, 100, and 120 mmHg.

The lipid and calcium components were modeled as incompressible Neo-Hookean solids with the strain energy density function, W_*Neo*−*Hookean*_, given as

(1) $${\text{W}}_{\mathrm{Neo}-\mathrm{Hookean}}=c_{1}\,\left(I_{1}-3\right)$$

where *I*_1_ is the first invariant of the right Cauchy-Green deformation tensor and *c*_1_ is the material constant. The *c*_1_ material constant was taken as 1 kPa (Loree et al., 1994; Akyildiz et al., 2011) and 1 GPa (Gijsen et al., 2021) for lipid and calcium, respectively. The fibrous intima and arterial wall components were modeled as incompressible Yeoh materials, where the strain energy density function W_Yeoh_ is to be given as

(2) WYeoh=∑i=13ci(I−13)i

where the material constants are indicated by *c*_i_ for *i* = 1, 2, 3. During the iFEM protocol, the Yeoh material constants (*c*_i_) of the fibrous intima and wall were optimized by iteratively predicting a total of six parameters.

### Optimization Scheme

In the last part of the iFEM scheme, the errors between the experimental and computationally derived axial displacements were minimized iteratively by varying the model constants’ values of the fibrous intima and the arterial wall in the FE model simulations.

#### Grid Application and Error Calculation

A grid with an element size of 100 μm × 100 μm was applied on the analyzed atherosclerotic artery cross-sections’ FE model and ultrasound displacement data. For the minimization, only the grid elements located in the mid-section, spanning the 50% of the cross-sectional width, were used for the error calculation in the optimization scheme (Figure 1). Within a grid element, the displacements of the FE nodes (∼ 10 FE nodes per grid element) and of the ultrasound data were averaged to obtain the experimental and the computational displacement vectors. These vectors contained displacement information from 80, 100, and 120 mmHg pressure steps. Then, the normalized mean square error (NMSE) (Torun and Swaminathan, 2019) given as

(3) $$\text{NMSE}=f\,\left(x\right)=\frac{\sum_{i=1}^{N}{\left(\hat{y}\,\left(x_{i}\right)-y\,\left(x_{i}\right)\right)}^{2}}{\sum_{i=1}^{N}{\left(y\,\left(x_{i}\right)-\frac{1}{N}\,\sum_{i=1}^{N}y\,\left(x_{i}\right)\right)}^{2}}$$

was calculated for the minimization, where *N* is the number of the selected grid elements, and y (*x*_i_) and $\hat{y}\,\left(x_{i}\right)$ are the vectors composed of the experimentally and computationally derived displacements, respectively.

#### Deep Partitioning Tree Based Bayesian Optimization

As for the optimization algorithm, a machine learning based global optimization technique called deep partitioning tree based BO (DPTBO) (Torun and Swaminathan, 2019) was used. The optimization part of the iFEM framework corresponds to a non-linear problem with many possible local minima, where each function evaluation involves a complex FE analysis. Hence, the optimization algorithm chosen needs to be able to avoid these local minima, while minimizing the number of function evaluations in order to locate the global minimum in a practical amount of time. The DPTBO and, in general, BO-based algorithms are specifically developed to handle such difficult problems. The sample efficiency of BO algorithms comes from utilizing a machine learning model that performs probabilistic non-linear regression, namely, a Gaussian process (GP). More specifically, at each iteration of BO, all the previously collected function evaluations are used to train a GP model that provides a predictive posterior distribution, which can “predict” the value of cost function without performing an actual function evaluation, in our case FE analysis, and provide a confidence interval around this prediction. The predicted value (posterior mean) and the confidence interval (posterior variance) are then used to create a “sampling strategy,” which is used to select the input parameters that provide the highest likelihood of being the global minimum. The selected sample then gets evaluated through an actual FE analysis, and the result is used to re-train the GP to proceed into next iteration. The interested readers are referred to Brochu et al. (2010) for a more detailed description of BO-based methods and their advantages over commonly used optimization methods.

Note that for all cases, we used 50 initial guesses and set the maximum number of model evaluations to 400 as the optimization was observed to converge well-before this pre-set number. After the BO procedure was completed, it provided the final GP model that can be used for post-processing. One particularly useful post-processing step of our iFEM approach is to evaluate the possible uniqueness of the predicted material properties. The uniqueness information can be obtained by “inverting” the GP model, i.e., asking it to provide a set of material properties that would give a similar cost function value as the actual result of optimization. In the current study, the similarity was defined as 5% + actual error. In case that the median of the set of material properties obtained using inverting the GP is similar to the actual result of iFEM, then this implies the likelihood of the uniqueness of the estimated parameters. This inversion of the final GP model is implemented in MATLAB (MathWorks, Portola Valley, CA, United States) using a technique called slice-sampling (Neal, 2003), which can be found in the open-source “GPstuff” package (Vanhatalo et al., 2013).

## Results

Ten cross-sections from five atherosclerotic human coronary arteries were characterized by the developed iFEM approach. The fibrous intima and arterial wall components in the cross-sections had an average area ± SD of 2.64 ± 1.45 and 4.61 ± 2.43 mm^2^, respectively. All cross-sections contained a lipid component with an average area of 1.33 ± 0.84 mm^2^. Three cross-sections (#7, 9, and 10) had calcifications with an average area of 0.40 ± 0.50 mm^2^ (Supplementary Table 1).

**FIGURE 4:**
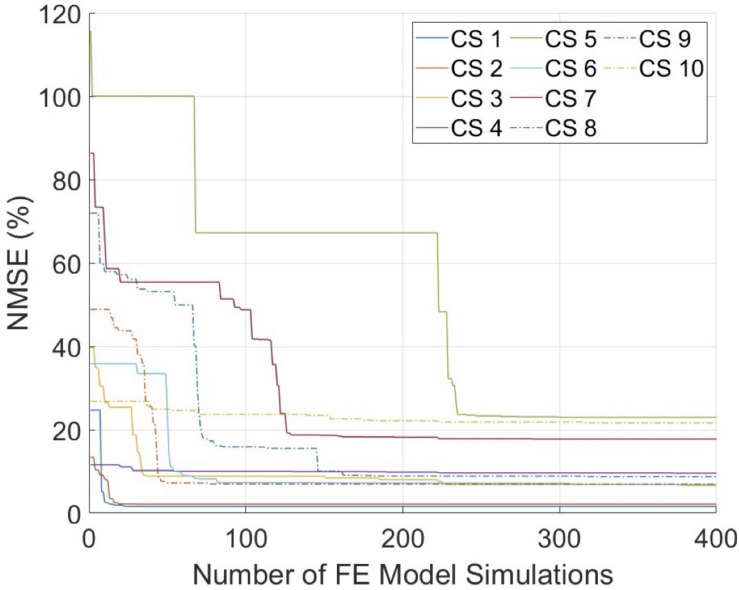
The convergence graphs of the inverse finite element modeling (iFEM) evaluations. CS, cross-section; FE, finite element; NMSE, normalized mean square error.

**FIGURE 5:**
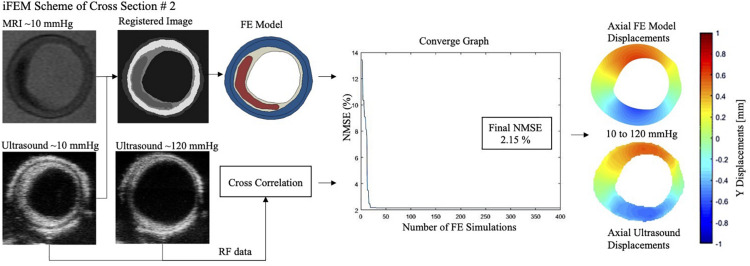
Magnetic resonance imaging (MRI), ultrasound image (at 10 and 120 mmHg), finite element (FE) model, convergence graph and experimental and computationally derived final displacement maps demonstrated for the representative cross-section #2 with an error of 2.15%. NMSE, normalized mean square error.

The mechanical inflation tests were performed successfully on all arteries with minimal or no leakage, and stabilized intraluminal pressure at each pressure step. For all cross-sections, the optimization scheme reached convergence within the pre-set budget of 400 iterations (Figure 4). The average error was 10.5%, where seven cross-sections had an error ≤10%. Figure 5 demonstrates the MRI and ultrasound images, FE model, convergence curve, and experimentally and computationally predicted displacement patterns of the cross-section #2, as a representative case.

The predicted Yeoh material constants and error values for all cross-sections are given in Table 2. The predicted material constants for the fibrous intima and wall components all satisfied the Yeoh material model stability criteria (Bilgili, 2004) given as

(4) $$c_{1}>0,-\sqrt{3\,c_{1}\,c_{3}}<c_{2}<\infty ,c_{3}>0$$

**FIGURE 6:**
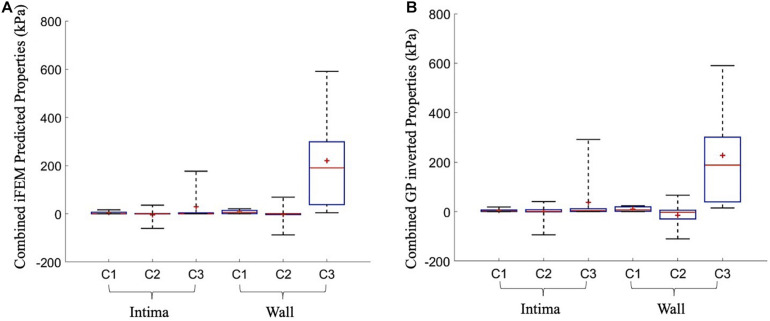
The overall predicted material constants by the inverse finite element modeling (iFEM) predicted **(A)** and the Gaussian process (GP) inverted **(B)** methods for 10 cross-sections.

The uniqueness of the iFEM predicted material properties was analyzed by inverting the GP model that is trained during the iFEM protocol. During the GP inverting process, additional set of material constants were predicted that converged in less than their iFEM finalized NMSE + 5% range. Then, they were compared with the iFEM predicted Yeoh constants for all of the 10 cross-sections. Figure 6 outlines the iFEM-predicted and the GP-inverted model predicted Yeoh model material constants for the fibrous intima and wall layers. For the fibrous intima, the medians of the material constants, namely, *c_1*, *c_2*, and *c_3*, were 1.34, 0.20, and 0.36 kPa for the iFEM-predicted, and 2.35, 1.24, and 4.09 kPa for the GP-inverted methods, respectively. For the wall layer, the medians of 5.90, −0.27, and 190.48 kPa were observed for the iFEM predicted constants, whereas the GP-inverted model had a median of 7.06, −2.39, and 188.37 kPa, respectively. This analysis shows good similarity between the iFEM-predicted and GP-inverted model results, implying the high likelihood of the uniqueness of the iFEM predicted values. Please note that the provided median values were estimated to predict the similarity between the iFEM-predicted and GP-inverted model results. The future FE models should not be based on these median values, as the large variation within the distributions clearly shows the importance of individual-specific characterization.

**FIGURE 7:**
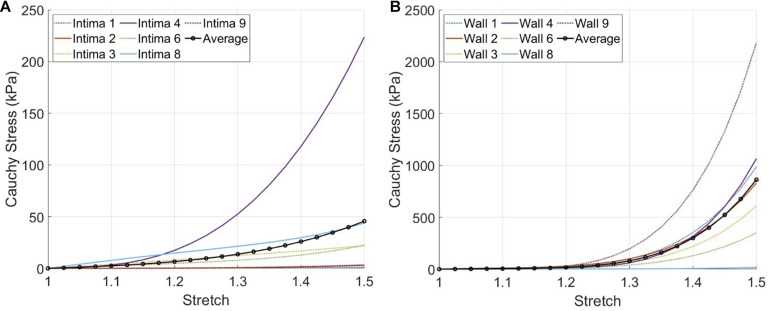
The expected material responses under uniaxial tensile stretching condition using the inverse finite element modeling (iFEM) predicted Yeoh constants for the fibrous intima **(A)** and wall **(B)** components of seven cross-sections, excluding the outlier cases #5,7, and 10.

Currently, there are a variety of mechanical tests that have been applied on atherosclerotic human arteries and material models that have been fitted to the experimental data available (Akyildiz et al., 2014). This complicates the direct comparison of material constants among the different studies. For an easy comparison of our results to other studies, we evaluated the Cauchy stress-stretch responses of the fibrous intima and wall components with our predicted material behavior under uniaxial tensile stretching condition, as this is the most commonly used testing technique (Figure 7). The average Cauchy stress results for 1.1, 1.2, and 1.3 stretch ratios were obtained as 2.5, 6.5, and 13.7 kPa for the fibrous intima, and 5.0, 17.0, and 80.0 kPa for the wall components, respectively. The fibrous intima ranged up to 7.7, 17.4, and 52.5 kPa for 1.1, 1.2, and 1.3 stretch ratios, whereas the wall ranged to 10.9, 29.2, and 195.9 kPa, respectively (Supplementary Table 2). [Note that we excluded for this analysis the three cases (#5, 7, and 10) associated with an optimization error >10%.]

## Discussion

The mechanical characterization of atherosclerotic human coronary arteries is crucial for accurate assessment of local plaque stress patterns toward the plaque rupture risk prediction. Current knowledge on the material properties of atherosclerotic human coronary arteries is limited. This is possibly due to the challenges including the difficulty in collecting atherosclerotic human coronary arteries, and the small size and the difficult anatomical features (e.g., side branches) of the arteries, which complicate mechanical testing. In the few studies that tested atherosclerotic human coronaries (Kural et al., 2012; Karimi et al., 2013a,b, 2017a,b; Jankowska et al., 2015), tensile tests were performed on arterial samples with all layers/components intact and hence, only the aggregate artery properties could be reported. However, the properties of the individual components are crucial for assessing the plaque stress distribution accurately. In this work, we assessed the material properties of the fibrous intima and arterial wall components of atherosclerotic coronary arteries.

Overall, both the fibrous intima and the arterial wall showed the expected non-linear, strain-stiffening behavior. The arterial wall was observed to show stiffer behavior than the fibrous intima component. For these two components, some cross-sections and arteries demonstrated very compliant behavior. Such variation in tissue behavior is possibly attributable to the difference in the microstructural composition of the tissues (Akyildiz et al., 2014). Similar variance was also observed for carotid plaques by Lawlor et al. (2011).

To the best of our knowledge, there is only one prior study that investigated the material behavior of the individual components of diseased human coronary arteries. In that study, Holzapfel et al. (2005) performed tensile testing on the dissected layers of intima, media, and adventitia. Their average circumferential Cauchy stress responses obtained for 1.1, 1.2, and 1.3 stretch ratios were approximately 5, 21, and 87 kPa for the media, and 5, 17, and 141 kPa for the adventitia. Their results for the media and adventitia components were similar with each other, and in accordance with our arterial wall results. Holzapfel et al. (2005) reported average circumferential Cauchy stress results of the non-atherosclerotic, thickened intima for 1.1 and 1.2 stretch ratios approximately as 21 and 120 kPa, respectively. Although the average intima tissue behavior given by Holzapfel et al. (2005) was stiffer than our results, there is an overlap of the tissue behavior ranges reported by the two studies.

In the developed iFEM framework, we used the inflation experiments as mechanical testing technique due to its benefits on achieving physiological-like testing conditions by; (1) the preservation of tissue integrity and (2) the application of physiological multiaxial loading. Besides the inflation testing, the developed pipeline comprised non-invasive imaging techniques of high-resolution MRI and ultrasound imaging, and a sample efficient machine learning-based optimization algorithm. Ultrasound imaging and measurements span the entire 3D structure of the arteries where the ultrasound probe attached to a linear motor swept the arteries in its longitudinal direction. However, it is important to note that the deformations were confined to transversal planes (axial–transversal plane of the ultrasound beam or radial–circumferential plane of the artery) as the arteries were tested under a constant longitudinal pre-stretch. This prevented any out-of-plane deformation in the longitudinal direction. The ultrasound data were also 2D, acquired on these transversal planes at multiple longitudinal locations/cross sections. Therefore, 2D FE models were used to simulate the mechanical tests. Atherosclerotic intima is a fibrous tissue, with a very disorganized fiber alignment (Akyildiz et al., 2017); hence, it does not exhibit a distinct global predominant fiber orientation. Therefore, in the 2D FE models, we used an isotropic material model instead of an anisotropic one in our study to describe the mechanical behavior of atherosclerotic coronary arteries. We chose to use Yeoh material model, as also used by others (Lawlor et al., 2011; Cunnane et al., 2015), due to its good performance to capture the non-linear mechanical behavior of atherosclerotic arteries (Teng et al., 2015). In addition, its availability in many FE modeling platforms provides an easy use in future clinical translations for non-expert users. The obtained error results with the average of 10.5% were also an indication of the combination of goodness of the Yeoh material model fitting and the ultrasound registered MRI geometry.

From the optimization perspective, the iFEM is a challenging problem. The optimization needs to be performed at “black-box” setting where the gradients are not available and the only way to get information about the function is through a computational simulation. The simulations were performed through FE method, which can lead to an impractical optimization time if a large number of simulations are performed. Hence, the optimization problem in iFEM corresponds to a potentially high-dimensional black-box optimization where the number of function evaluations needs to be minimized. BO-based methods, such as DPTBO, are specifically developed to handle such problems. Here, the use of GPs allows to incorporate predictive information about the objective function into the optimization strategy, which leads to minimizing the number of simulations required for convergence. Such predictive information is not available in more traditional methods such as the grid search, genetic algorithm, and differential evolution, making BO-based methods superior to these for iFEM purposes. In addition, the trained GP models hold the advantage of being inverted to gain knowledge regarding the uniqueness assessment of the iFEM predicted material constants, which is a great advantage compared to the traditional optimization methods.

The developed framework has some limitations. (1) The atherosclerotic human coronary arteries are rare and difficult to collect. Therefore, the number of coronary samples used in the studies (Baldewsing et al., 2005; Kural et al., 2012; Karimi et al., 2013a,b, 2017a,b; Jankowska et al., 2015) that aimed the mechanical characterization has been always limited. The same limitation holds for our current study as well where we tested five atherosclerotic human coronary arteries. (2) The mechanical characterization was performed following a freezing protocol rather than using fresh samples. However, the effect of storage at −80°C is not expected to affect the mechanical properties, as shown by Schaar et al. (2002). (3) The artery was constrained in the longitudinal direction during the inflation testing. Hence, the displacements occurred only in the 2D plane, in axial and lateral directions. The iFEM analysis was only based on axial displacements. Clearly, one would prefer to utilize the full 2D displacement field for the analysis. However, as the ultrasound displacement measurements in the lateral direction was shown to have lower signal-to-noise ratio (SNR) than the ones in the axial direction (Akyildiz et al., 2016), we have decided to utilize only the high SNR data in the axial direction. In addition, mid-section region, spanning the 50% width of the cross-section, was selected as the grid application location due to decreasing SNR caused by small axial displacements in the side regions (Akyildiz et al., 2016). The impact of utilizing the full 2D displacement field on the results warrants further research. (4) The tissue components in the FE models were assumed to be isotropic. Although the error values were reasonably low, implying a good representation of the material behavior by the isotropic Yeoh models, the anisotropy should be addressed in future work for more accurate material characterization. (5) The geometry for the FE models was based on the 20% pre-stretched reference configuration. Sommer et al. (2018) observed that the deformation change on the transversal cross-sections due to the pressure increase in non-pre-stretched and 20% pre-stretched iliac arteries was very similar; hence, the longitudinal pre-stretch of the arteries in the tests was not incorporated in the FE models. We do not expect any major differences in our results if we had incorporated the longitudinal pre-stretch since the input in our iFEM approach was the pressure increase and the corresponding change in deformation in the transversal plane. (6) Assessment of residual stresses is very challenging for atherosclerotic plaques due to their heterogenous structure. The opening angle method, commonly used for healthy arteries (Chuong and Fung, 1986; Fung, 1991; Delfino et al., 1997), was proposed for the assessment of the residual stresses in atherosclerotic arteries (Ohayon et al., 2007). However, if a single cut can release the residual stresses completely in the complex structure of atherosclerotic plaques is still not clear. As there is yet no well-established means of assessing residual stresses in atherosclerotic arteries, we did not incorporate them in the current study.

The iFEM framework developed in this study has the potential for clinical application. The approach can be utilized for both coronary and carotid arteries. For carotid arteries, clinical counterparts of the MRI and ultrasound systems employed in this study can be used for the *in vivo* assessment of morphological information and deformation, respectively. Yet, the feasibility and performance of the iFEM approach when combined with these clinical imaging modalities require to be further explored. For coronary arteries, although the smaller vessel size and the heart motion pose extra challenges in the clinical translation, the recent advancements in MRI are promising for coronary imaging (Makowski and Botnar, 2013; Dweck et al., 2016). An important advantage of the developed iFEM framework is that it allows other imaging modalities to be incorporated, including intravascular ultrasound (De Korte et al., 2000, 2002) and the optical coherence tomography (Kok et al., 2016), which are currently used in clinical settings for coronary arteries for morphological information. The intravascular ultrasound also has the capability to measure deformations in coronary arteries (Veress et al., 2002).

## Conclusion

The material property information of atherosclerotic human coronary arteries is crucial for accurate mechanical analyses of the arteries. However, this information is currently very scarce, and limited to aggregate, average artery properties. This work presented, for the first time, the multicomponent atherosclerotic human coronary material properties obtained from physiological-like mechanical testing, i.e., inflation experiments, allowing intact tissue testing. To reach this goal, we developed an advanced iFEM approach, which utilized a sample efficient optimization and high-resolution MRI and ultrasound systems. The developed framework enabled us characterizing the mechanical behavior of the fibrous intima and the arterial wall of atherosclerotic coronary arteries.

## Data Availability Statement

The original contributions presented in the study are included in the article/Supplementary Material. Further inquiries can be directed to the corresponding author/s.

## Ethics Statement

The studies involving human participants were reviewed and approved by the Medical Ethics Committee of Erasmus Medical Center in Rotterdam. The patients/participants provided their written informed consent to participate in this study.

## Author Contributions

SG performed the computational modeling, developed the numerical technique, analyzed the data, and prepared the manuscript. HT developed the optimization technique, implemented the numerical pipeline, and prepared the manuscript. HH and CK developed the technique for ultrasound-derived tissue deformation measurements. HH contributed to the preparation of the manuscript. GG performed the image segmentation and registrations and assisted the computational modeling. IB and VC performed the mechanical experiments and tissue imaging. AS and FM provided feedbacks and critically revised the manuscript. CC and AA contributed to the study design, supervised the study, and reviewed the manuscript. AA was the primary supervisor of the project. All authors contributed to manuscript revision, read, and approved the submitted version.

## Conflict of Interest

The authors declare that the research was conducted in the absence of any commercial or financial relationships that could be construed as a potential conflict of interest.

## Publisher’s Note

All claims expressed in this article are solely those of the authors and do not necessarily represent those of their affiliated organizations, or those of the publisher, the editors and the reviewers. Any product that may be evaluated in this article, or claim that may be made by its manufacturer, is not guaranteed or endorsed by the publisher.
