# Supplementary material for: Multicomponent Mechanical Characterization of Atherosclerotic Human Coronary Arteries: An Experimental and Computational Hybrid Approach
Source: Front Physiol. 2021 Sep 7;12:733009. doi: 10.3389/fphys.2021.733009 (PMC8452922; doi:10.3389/fphys.2021.733009)
Supplement: Supplementary file 1 [file Data_Sheet_1.PDF]

Supplementary Material

Supplementary Table I. Area measurements of the components [mm^2] (CS: cross section, SD: standard deviation)

	Fibrous Intima	Lipid	Calcification	Wall
CS 1	3.30	0.84	-	4.74
CS 2	1.74	1.40	-	4.96
CS 3	2.60	0.72	-	6.60
CS 4	2.36	1.05	-	1.91
CS 5	2.13	1.03	-	4.30
CS 6	1.56	1.47	-	2.90
CS 7	1.42	0.44	0.09	2.18
CS 8	1.03	0.71	-	2.24
CS 9	5.27	2.83	0.14	7.09
CS 10	4.94	2.80	0.97	9.20
Average	2.64	1.33	0.40	4.61
SD	1.45	0.84	0.50	2.43

Supplementary Table II. The Cauchy Stress results of fibrous intima and wall components at 1.1, 1.2 and 1.3 stretch ratios. Please note that we excluded the three cross sections (#5, 7, and 10) associated with an optimization error > 10%) for the average calculation. (CS: cross section, SD: standard deviation)

CS Number	Stretch 1.1		Stretch 1.2		Stretch 1.3	
	Fibrous Intima	Wall	Fibrous Intima	Wall	Fibrous Intima	Wall
CS 1	0.06	4.14	0.14	19.88	0.26	97.59
CS 2	0.08	9.54	0.23	29.20	0.58	99.73
CS 3	4.00	4.53	8.09	15.30	12.36	63.02
CS 4	3.17	10.86	17.39	12.89	52.50	61.33
CS 5	0.54	8.49	1.87	18.29	4.72	35.68
CS 6	2.35	3.51	4.77	10.95	7.83	39.66
CS 7	0.24	4.16	0.69	37.27	1.59	150.66
CS 8	7.71	1.11	14.71	1.86	21.37	2.92
CS 9	0.08	1.45	0.29	28.90	0.76	195.88
CS 10	8.56	0.28	10.00	0.76	37.06	1.93
Average	2.5	5.0	6.5	17.0	13.7	80.0
SD	2.8	3.8	7.2	9.9	18.8	61.0
